# DETECTION OF OCCULT LYMPH NODE TUMOR CELLS IN NODE-NEGATIVE GASTRIC CANCER PATIENTS

**DOI:** 10.1590/0102-6720201700010009

**Published:** 2017

**Authors:** Marina Alessandra PEREIRA, Marcus Fernando Kodama Pertille RAMOS, Andre Roncon DIAS, Osmar Kenji YAGI, Sheila Friedrich FARAJ, Bruno ZILBERSTEIN, Ivan CECCONELLO, Evandro Sobroza de MELLO, Ulysses RIBEIRO-JR

**Affiliations:** 1 Hospital das Clínicas; 2 Cancer Institute, University of São Paulo Medical School, São Paulo, SP, Brazil.

**Keywords:** Gastric cancer, Micrometastasis, Lymph node metastasis, Immunohistochemistry.

## Abstract

**Background:**

The presence of lymph nodes metastasis is one of the most important prognostic indicators in gastric cancer. The micrometastases have been studied as prognostic factor in gastric cancer, which are related to decrease overall survival and increased risk of recurrence. However, their identification is limited by conventional methodology, since they can be overlooked after routine staining.

**Aim:**

To investigate the presence of occult tumor cells using cytokeratin (CK) AE1/AE3 immunostaining in gastric cancer patients histologically lymph node negative (pN0) by H&E.

**Methods:**

Forty patients (T1-T4N0) submitted to a potentially curative gastrectomy with D2 lymphadenectomy were evaluated. The results for metastases, micrometastases and isolated tumor cells were also associated to clinicopathological characteristics and their impact on stage grouping. Tumor deposits within lymph nodes were defined according to the tumor-node-metastases guidelines (7^th^ TNM).

**Results:**

A total of 1439 lymph nodes were obtained (~36 per patient). Tumor cells were detected by immunohistochemistry in 24 lymph nodes from 12 patients (30%). Neoplasic cells were detected as a single or cluster tumor cells. Tumor (p=0.002), venous (p=0.016), lymphatic (p=0.006) and perineural invasions (p=0.04), as well as peritumoral lymphocytic response (p=0.012) were correlated to CK-positive immunostaining tumor cells in originally negative lymph nodes by H&E. The histologic stage of two patients was upstaged from stage IB to stage IIA. Four of the 28 CK-negative patients (14.3%) and three among 12 CK-positive patients (25%) had disease recurrence (p=0.65).

**Conclusion:**

The CK-immunostaining is an effective method for detecting occult tumor cells in lymph nodes and may be recommended to precisely determine tumor stage. It may be useful as supplement to H&E routine to provide better pathological staging.

## RESUMO

**Racional:**

A presença de metástase em linfonodos é um dos indicadores prognósticos mais importantes no câncer gástrico. As micrometástases têm sido estudadas como fator prognóstico no câncer gástrico, sendo relacionadas à diminuição da sobrevida global e aumento do risco de recidiva da doença. Entretanto, sua identificação é limitada pela metodologia convencional, uma vez que podem não ser identificadas pela rotina histopatológica por meio da coloração de H&E.

**Objetivo:**

Investigar a presença de células tumorais ocultas através de imunoistoquimica utilizando as citoqueratinas (CK) AE1/AE3 em pacientes com câncer gástrico com linfonodos histologicamente classificados como negativos por H&E.

**Métodos:**

Quarenta pacientes (T1-T4N0) submetidos à gastrectomia potencialmente curativa com linfadenectomia D2 foram avaliados. A presença de metástases, micrometástases e células tumorais isoladas foram correlacionadas com características clínicopatológicas e impacto no estadiamento. Os depósitos tumorais nos linfonodos foram classificados de acordo com o sistema TNM (7º TNM).

**Resultados:**

Um total de 1439 linfonodos foi obtido (~36 por paciente). Células tumorais foram detectadas por imunoistoquimica em 24 linfonodos de 12 pacientes (30%). As células neoplásicas estavam presentes na forma isolada ou em cluster. Invasão tumoral (p=0,002), venosa (p=0,016), linfática (p=0,006) e perineural (p=0,04), assim como resposta linfocítica peritumoral (p=0,012) foram correlacionadas com linfonodos CK-positivos que originalmente eram negativos à H&E. Dois pacientes tiveram o estadiamento alterado, migrando do estádio IB para IIA. Quatro dos 28 CK-negativos (14,3%) e três dos 12 CK-positivos (25%) tiveram recorrência da doença (p=0,65).

**Conclusão:**

A imunoistoquimica é meio eficaz para a detecção de células tumorais ocultas em linfonodos, podendo ser recomendada para melhor determinar o estágio do tumor. Ela pode ser útil como técnica complementar à rotina de H&E, de modo a fornecer melhor estadiamento patológico.

## INTRODUCTION

Lymph node metastasis is considered one of the most important prognostic indicators in gastric carcinoma (GC), thus the number of positive lymph node (LN) is essential to stratify patients by stages and may useful for predicting patient survival^3^ ^,^ ^16^ ^,^ ^25^ ^,^ ^29^ ^,^ ^30^ ^,^ ^31^. Surgery with complete tumor removal, adequate free margins and regional lymphadenectomy is the best treatment option for resectable GC. It has been widely accepted that the procedure provides the best results in reduction of locoregional tumor recurrences and improvement of survival in patients with GC^11^ ^,^ ^28^.

Nevertheless, despite curative resection of their primary tumor, some patients with histologically node negative (pN0) GC based on conventional histological H&E staining still have local or distant tumor recurrence^11^ ^,^ ^17^. Occult lymph node micrometastasis (LNmi) within regional LNs that is not detected by routine histological examination has been suspected to be a key causative factor of recurrence and metastasis in these patients^17^ ^,^ ^32^.

The aim of the present study was to identify the incidence of potentially relevant occult tumor cells in patients with pN0 GC using cytokeratins immunostaining, and analyze their impact on stage grouping. The results for the presence of metastasis, micrometastasis and tumor deposits were also associated with clinicopathological characteristics.

## METHODS

### Patients

This study was approved by our institution ethics committee, and all patients signed the informed consent form before their inclusion in the study (CEP-FMUSP: 354/13).

Were retrospectively analyzed patients submitted to potentially curative resection with D2 lymphadenectomy for GC between 2009 and 2014 at Cancer Institute of University of São Paulo, São Paulo, SP, Brazil. The inclusion criteria were: radical gastrectomy with D2 lymphadenectomy, R0 resection, node-negative patients (pN0) by H&E histopathologic routine examination, absence of distant metastasis, adequate archival tissue for analysis and the availability of complete clinicopathologic data.

Clinicopathological characteristics about tumor and disease recurrence were collected from the hospital gastric cancer data bank.

All appraisal cases samples were obtained surgically from patients at the gastrointestinal surgery service and were histopathologically examined.

### Anatomopathological evaluation 

Primary tumors and LNs were fixed in 10% formalin solution and embedded in paraffin. Processing of the resection specimens was done using a standardized protocol. All LN were routinely stained with H&E and were classified as pN0. For histological examination and diagnostic confirmation of selected cases, the original H&E sections of all LNs were re-analysed by pathologists from the service.

The presence or absence of LNmi was examined using a representative sections obtained from the total inclusion of nodal structure.

### Detection of tumor cells by immunohistochemistry

The LNs from pN0 cases were re-evaluated by immunohistochemistry (IHC) using antibodies against human cytokeratins (CK) AE1/AE3 (Dako Corporation, Carpinteria, CA 93013 USA) in order to identify the cytokeratins of malignant cells inside LNs. The antibody cocktail AE1/AE3 is specific for a range of human cytokeratins in epithelial cells and does not react with lymphoid tissue.

Tissue sections from paraffin blocks were deparaffinized with xylene and rehydrated with graded ethanol dewaxed. After, the histologic sections were subjected to heat-induced antigen retrieval with citrate buffer solution (pH 6.0) in a steamer for 30 min. Endogenous peroxidase was blocked with H_2_O_2_ (6%), and sections were incubated overnight with primary antibodies at 4° C. The secondary antibody was applied and followed by the application of peroxidase-labeled streptovidin (Novolink - Novocastra Laboratories, Newcastle, UK). The reaction products were visualized with diaminobezidine as the chromogen and sections were counterstained with Harris's hematoxylin. Tris-buffered saline was used as a negative control instead of primary antibody for negative controls.

Microscopic analysis was carried out using a conventional light microscope. Dark brown insolvable precipitate was formed peripherically around malignant cells, corresponding to cytokeratin-positive cells. The CK AE1/AE3-positive cells in the LNs were compared with the same sections stained with H&E.

### Definition of lymph node positive findings

Tumor deposits within LNs were defined and classified according to the 7^th^ edition of the TNM guidelines. The distinction among metastasis, micrometastasis and isolated tumor cells (ITC) was based on the size of the metastastatic tumor foci.

Positive LN metastasis was defined when tumor cells measuring more than 2.0 mm were found. Micrometastasis was defined when tumor cells clusters measures between 0.2-2.0 mm. Metastasis or micrometastasis detected by morphological techniques, such as H&E staining or immunohistochemistry, should be classified as pN1 and pN1 micrometastasis, respectivelly, and included in the disease staging. ITC are single tumor cells or small clusters of cells measuring <0.2 mm in greatest dimension. Patients with ITC in LN are staged as pN0(i+), but do not change the TNM stage. Patients with some LN tumor deposits (micrometastasis, ITC or cluster) were defined as a "CK-positive" and patients without tumors cells in LN were defined as "CK-negative" for comparison analysis

### Statistical analysis

The clinicopathological variables of cases (CK-positive group) and controls (CK-negative group) were compared using the Fisher's exact test or Chi-square test. All tests were two-sided and a p-value less than 0.05 was considered statistically significant. Statistical analysis was performed using SPSS software, version 18.0 (SPSS Inc, Chicago, IL).

## RESULTS

Were reviewed 255 GC cases and 111 patients were classificated as pN0. The mean age of the patients was 64.6 years (26-84), and the male/female ratio was 1.5 (3:2). A total of 1439 LNs was obtained. The average number of LNs dissected per patient was 36. Among these 111 cases, a consecutive series of 40 patients were included in this study. The cases were selected as follows: 11 pT1, 11 pT2, 11 pT3 and 7 pT4 tumors. All original H&E slides were reviewed and pN0 were confirmed by two distinct pathologists.

### Detection of lymph node tumor cells

**FIGURE 1:**
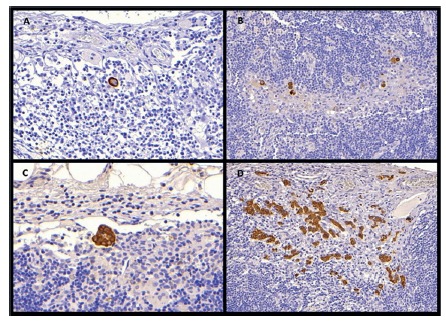
Cytokeratin (AE1/3) immunostaining in lymph nodes: A) isolated tumor cell (ITC); B) multiple cancer cells in the form of ITC; C) tumor cells in the form of clustered cancer cells; D) micrometastasis in the form of multiple cluster cells

A total of 29 LNs obtained from 12 (30%) patients contained tumor cells that were immunopositive for CK by immunohistochemistry staining. Two patients were pT2, five pT3 and five pT4. Micrometastasis were detected in three nodes from two pT2 patients (5%). The number of LNs involved ranged from 1-9 per patient, with an average of 2.4. In addition, it was seen among CK-positive group that the microinvolvement occurred as single-cell type in nine patients and as cell cluster-type in all 12 patients (Figure 1).

Two patients (T2 stage) with GC diagnosed as free of metastasis by ordinary H&E staining were re-staged because had evidence of micrometastasis in their regional LNs on the basis of immunohistochemistry analysis, migrating from stage IB to IIA.

### Occult tumor cells vs. clinicopathological parameters

No significant difference was noted between CK-positive and CK-negative groups in the analysis of clinicopathological data, including: patient age, gender, tumor location, histological type and average number of dissected LNs. Although there was no significant relationship between tumor size, the average of CK-positive group (4.0 cm) was larger than negative group (2.8 cm)

Lymph node CK positive was significantly associated with tumor depth of wall invasion (p=0.002), venous (p=0.016), lymphatic (p=0.006) and perineural invasions (p=0.04) and peritumoral lymphocytic response (p=0.012). The incidence of LNs tumor cells according to tumor depth was as follows: 0% in pT1, 18% pT2, 45% pT3, 71% pT4. Two CK-positive patients had neoadjuvant therapy. Table 1 shows the clinicopathologic characteristics of patients with LN CK-positive and CK-negative.

**TABLE 1:** Clinicopathologic characteristics of 40 GC patients pN0 by H&E with and without CK-positive tumor cells in LN by immunostaining

Variable	CK-negative	CK-positive	p
	n= 28 (%)	n=12 (%)	
Gender			0.72
Female	12 (49.9)	4 (33.3)	
Male	16 (57.1)	8 (66.7)	
Tumor location			0.14
Proximal	8 (28.6)	2 (16.7)	
Middle	9 (32.1)	3 (25)	
Distal	10 (35.7)	4 (33.3)	
Anastomosis	0 (0)	2 (16.7)	
NA	1 (3.6)	1 (8.33)	
Size of the lesion			0.18
Mean (cm)	2.8	4.0	
< 4.5 cm	18 (64.3)	5 (41.7)	
≥ 4.5 cm	10 (35.7)	7 (58.3)	
Borrmann type			0.38
I	5 (17.9)	0 (0)	
II	3 (10.7)	1 (8.33)	
III	17 (60.7)	10 (83.3)	
IV	1 (3.6)	1 (8.33)	
Undetermined	2 (7.2)	0 (0)	
Lauren´s classification			1
Intestinal-type	17 (60.7)	7 (58.3)	
Diffuse-type	9 (32.1)	4 (33.3)	
NA	2 (7.2)	1 (8.33)	
Grade of histological differentiation			1
G1/G2 - well/moderate differentiated	13 (46.4)	6 (50)	
G3 - poorly differentiated	15 (53.6)	6 (50)	
Depth of invasion			0.355
Early	11 (39.3)	0 (0)	
Advanced	17 (60.7)	12 (100)	
Pathologic T stage			0.002
T1 / T2	20 (71.4)	2 (16.7)	
T3 / T4	8 (28.6)	10 (83.3)	
Number of LN evaluated			
Mean	36.5	34.7	0.41
≤ 25	5 (17.9)	4 (33.3)	
> 25	23 (82.1)	8 (66.7)	
Venous invasion			0.016
Absent	23 (82.1)	5 (41.7)	
Present	4 (14.3)	7 (58.3)	
NA	1 (8.33)	0 (0)	
Lymphatic invasion			0.006
Absent	20 (71.4)	3 (25)	
Present	7 (25)	9 (75)	
NA	1 (8.33)	0 (0)	
Perineural invasion			0.040
Absent	19 (67.8)	4 (33.3)	
Present	8 (28.6)	8 (66.7)	
NA	1 (8.33)	0 (0)	
Lymphocytic peritumoral response			
Mild / nearly absent	9 (32.1)	9 (75)	0.012
Moderate / Intense	17 (60.7)	2 (16.7)	
NA	2 (7.2)	1 (8.33)	
Neoadjuvant chemotherapy			0.209
Yes	1 (8.33)	2 (16.7)	
No	27 (96.4)	10 (83.3)	
Disease Recurrence			0.4
Yes	4 (14.3)	3 (25)	
No	24 (85.7)	9 (75)	

NA=not available

The median length of postoperative follow-up was 33 months, ranging from 2-72 months. During the follow-up period, a total of seven cases of cancer recurrence were reported. Although not statistically significant, the recurrence was more common in CK-positive patients (3/12, 25%) than in CK-negative group (4/28, 14.3%). Of the three CK-positive patients with disease recurrence, one was pT3 and two were pT4. In all of them only tumor cluster-type and single-cell were observed. 

## DISCUSSION

Lymph node metastasis is known as an important prognostic factor in GC, once the curative surgery with LN dissection admittedly reduces the risk of recurrence^17^. For this reason, studies have been conducted in order to improve the "N" stage evaluation, including implementation of "lymph nodes revealing solution" to increase the amount of retrieved nodes and the addition of complementary methods for tumor cells detection in the histopathological diagnostic routine^7^ ^,^ ^8^.

The histologic examination using H&E staining is the gold-standard for lymph node metastasis diagnosis. However, sometimes the LN status determined through conventional histological methods does not adequately reflect the prognosis^15^. The routine method of serial sections can miss gastric tumor cells due the heterogeneous distribution of metastatic foci avoiding the examination to access the full depth of LN involvement by the neoplasia^2^ ^,^ ^21^ ^,^ ^22^ Consequently, recurrence can occur in node negative patients, and it is possible that such disease relapse originates from otherwise undetected micrometastic cancer cells. Maehara et al.^17^ was one of the first researchers to highlight that even after curative resection of an early gastric cancer, some patients have recurrence, because they have occult micrometastasis in perigastric LNs at the time of original diagnosis^17^.

In this retrospectively designed study, were performed immunohistochemistry staining for cytokeratin (CKAE1/AE3) on LNs that remained negative for macrometastasis based on conventional H&E staining to evaluate the incidence of LN involvement in pN0 GC and their impact on stage grouping, determining whether other clinicopathologic parameters might be associated with their occurrence. Cytokeratin (CK) is a basic component of the cytoskeleton of epithelial cells, and they are reliably expressed by tumor cells. Through the use of specific antibodies, immunohistochemistry staining may facilitates the detection with the advantage that it can morphologically identify a single cancer cell or clusters of cancer cells that could readily be missed by routine histological examination^27^ ^,^ ^31^. Moreover, the sections depth from paraffin block for performing immunohistochemistry can make the tumor deposit more evident in the LNs. This method is useful especially in the Lauren's diffuse type, where it is difficult to detect when only a small number of tumor cells are present in the LN. Furthemore, compared with nodal metastases detected by H&E staining, micrometastasis detected by immunohistochemistry in most cases manifest as discrete or small clustered cancer cells in the medullar and marginal sinus of the LN^4^ ^,^ ^5^.

In contrast to immunohistochemistry, molecular markers allow analysis of the entire LN in one reaction, thus reducing the time needed for screening. Recent studies with GC demonstrated an increase in the detection of LNmi using reverse transcription-polymerase chain reaction (RT-PCR) assays for carcinoembryonic antigen or CK20 messenger RNAs^13^. However, false-positive results are allegedly common, since the specificity might be reduced by illegitimate expression of the respective marker gene from normal LN cells^23^. 

According to the 7^th^ TNM classification, LNmi detected by morphological techniques, such as H&E staining or immunohistochemistry, should be included in the staging of disease. In this sense, it is important to note that while many studies basically define micrometastasis as the presence of a single ITC or small cluster of gastric tumor cells identified by immunohistochemistry and not visualized by H&E, this study examined the incidence of LNs tumor cells on the basis of the TNM criteria, according to the size of the tumor deposit. In the present study, 12 cases (30%) with occult tumor cells were identified among 40 surgically treated pN0 GC. This frequency is consistent with those reported in the literature, where undetected tumor cells corresponding to 10-36% of pN0 patients^22^.

Generally, the presence of LN tumor cells has been associated to particular clinicopathological characteristics. Patients with more advanced pT category tend to have a higher incidence of micrometastasis compared with pT1N0 tumors^17^. Micrometastasis detected by immunohistochemistry were reported to occur in 10.7% of T1^20^ and in 20% of T2-T4^31^ of previous node-negative GC patients. In this series, all T1 patients had LNs CK-negative, while 41% of T2-T4 had LNs CK-positive. The present investigation showed that, besides the depth of wall invasion, LN occult involvement was significantly associated with advanced disease stage, lymphocyte peritumoral response, perineural and lymphovascular invasion, similar to other studies in the literature. Reports of micrometastasis incidence of 66.7% and 26.8% for patients with and without lymphatic invasion, respectively, reveal a significantly higher incidence of this characteristic in this group of patients^2^ ^,^ ^4^ ^,^ ^5^ ^,^ ^7^.

 While some authors suggest micrometastasis association with tumor size, grade of histological differentiation (G2/G3) and Lauren´s diffuse histology^4^ ^,^ ^5^ ^,^ ^11^ ^,^ ^12^ ^,^ ^26^, these results didn´t find any differences in LN involvement with respect to these characteristic. However, was observed that in the CK-positive group tumor tends to be larger than in the CK-negative group (4.0 vs. 2.8 cm).

Interesting, although neoadjuvant chemotherapy is able to eradicate the occult disease before surgery^10^, two of three patients with neoadjuvant therapy had CK-positive LNs, showing that chemotherapy or radiotherapy may not necessarily eliminate the micrometastasis. 

In this study, was found CK-positive tumor cells in 29 of the 1439 perigastric LN. The incidence of nodal involvement increased to 30% by immunohistochemistry with CKAE1/3, and 5% of patients which had previously been classified as pN0 by conventional H&E stain were re-staged to had evidence of micrometastasis in their regional LNs on the basis of immunohistochemistry analysis (stage IB to IIA). Although LNmi change the stage of the disease, one of the main points of debate still concerns to their clinical significance.

Recent studies have been conducted focusing on the significance and clinical importance of micrometastasis detection. In general, the presence of disseminated tumor cells in "tumor-free" lymph nodes has been associated with a poorer postoperative prognosis. However, there is no consensus as to the impact of LNmi on survival of patients with GC^2^ ^,^ ^12^ ^,^ ^15^ ^,^ ^22^.

Most authors agree that there is significant difference in prognostic and survival among patients with and without LNmi^2^ ^,^ ^7^ ^,^ ^17^ ^,^ ^26^ ^,^ ^31^. Yasuda et al.^31^ demonstrated that LNmi is an independent prognostic indicator for T2-T3pN0 GC and the number and level of LNmi were strongly associated with survival time (66% vs. 95%)^31^. A significantly lower survival rates was also observed by Dell'Aquila Jr et al.^7^, that found micrometastasis in 15 of 28 T1-T4N0 CG patient (2-year survival rate, 21,5% vs. 62,9%), showing that micrometastasis are an important risk factor for recurrence in GC, in a context of radical D2 lymphadenectomy^7^. Simillarly, Ru et al.^26^ results showed that the recurrence rate was significantly higher in the micrometastasis group than in the non-micrometastasis group^26^.

However, others reports support the hypothesis that micrometastasis detection by immunohistochemistry would not offer a significant benefit over conventional pathologic in stratifying patients for planning appropriate adjuvant therapy and for prognostic grouping in clinical stages^6^ ^,^ ^9^ ^,^ ^19^ ^,^ ^20^. Whether these detected cancer cells can proliferate or if they can be removed by the host's immune response is still unknown^4^ ^,^ ^5^ ^,^ ^22^. Morgagni et al.^19^ ^,^ ^20^ found no prognostic relevance in micrometastasis detected by immunohistochemistry in T1N0 tumors (89% vs. 94%), even in a long postoperative follow-up of 10 years. Furthermore, the clinical evaluation of LNmi is somewhat difficult, because of the differences in study designs and methods of detection, such as the sample size, tumor stage of patients, number of retrieved LNs based on the area of LN dissection, use of immunohistochemistry or other methods, types of antibodies used and, principally, the definition of micrometastasis ^9^ ^,^ ^22^ ^,^ ^29^.

Some studies have investigated the role of LNmi in the adoption of adjuvant therapies and, especially, its clinical impact in applying minimally invasive treatment. An accurate intraoperative diagnosis of occult LN involvement would be essential particularly in defining criteria for limited surgical dissection, including endoscopic mucosal resection or endoscopic submucosal dissection, and sentinel node navigation surgery. Micrometastasis diagnosis may help to guide the area of appropriate LN dissection, allowing a tailored lymphadenectomy for the patient. Additionally, a better diagnostic definition of micrometastasis may also help to distinguish the category of pN0 (micrometastasis +) patients with potential benefit of postoperative adjuvant therapy^1^ ^,^ ^2^ ^,^ ^11^ ^,^ ^15^ ^,^ ^18^ ^,^ ^23^.

In this study, was found a higher incidence of recurrence of the disease in CK-positive group than in the CK-negative group (25% vs. 14.3%), but this was not statistically significant. This finding may be justified due to the low number of patients involved in the study with a short period of follow-up. These initials results encourage our group to extend the analyses to a larger number of patients to better clarify the clinical significance of CK-positive LNs in GC patients. 

## CONCLUSION

The CK-immunostaining is an effective method for detecting occult tumor cells in lymph nodes and may be recommended as supplement to H&E routine to refine pathological staging in GC. Due to its association with characteristics related to a worse prognosis, the identification of tumor cells in lymph nodes may be useful as one additional information for follow-up and risk factor for recurrent gastric cancer.
